# Association of Lesion Location and Functional Parameters with Vision-Related Quality of Life in Geographic Atrophy Secondary to Age-related Macular Degeneration

**DOI:** 10.1016/j.oret.2024.01.025

**Published:** 2024-02-03

**Authors:** Sandrine H. Künzel, Eliza Broadbent, Philipp T. Möller, Moritz Lindner, Lukas Goerdt, Joanna Czauderna, Steffen Schmitz-Valckenberg, Frank G. Holz, Maximilian Pfau, Monika Fleckenstein

**Affiliations:** 1 Department of Ophthalmology, University of Bonn, Bonn, Germany.; 2 Department of Ophthalmology & Visual Sciences, John A. Moran Eye Center, University of Utah, Salt Lake City, Utah.; 3 Department of Neurophysiology, Institute of Physiology and Pathophysiology, Philipps University, Marburg, Germany.; 4 Nuffield Laboratory of Ophthalmology, Sleep and Circadian Neuroscience Institute, Nuffield Department of Clinical Neurosciences, University of Oxford, Oxford, United Kingdom.; 5 Institute of Molecular and Clinical Ophthalmology Basel (IOB), Basel, Switzerland.

**Keywords:** Age-related macular degeneration, Geographic atrophy, Patient-reported outcomes, Quality of life, Vision-related quality of life

## Abstract

**Objective:**

The primary goal of this study was to determine how structural and functional parameters influence the vision-related quality of life (VRQoL) in patients suffering from geographic atrophy (GA) secondary to age-related macular degeneration.

**Design:**

This study was designed as a prospective, noninterventional, natural-history study (Directional Spread in Geographic-Atrophy study, NCT02051998).

**Subjects:**

The research involved 82 patients with bilateral GA.

**Methods:**

The study examined parameters including GA location as assessed by the ETDRS grid, best-corrected visual acuity, low-luminance visual acuity (LLVA), reading acuity, and speed. These parameters were then correlated with VRQoL, which was gauged using the National Eye Institute Visual Function Questionnaire 25. The analysis method employed was the least absolute shrinkage and selection operator with linear mixed-effects models.

**Main Outcome Measures:**

The central parameters measured in this study encompassed GA area, VRQoL scores associated with different GA subfields, and the significance of LLVA for foveal-sparing patients.

**Results:**

On average, patients showed a total GA area of 2.9 ± 1.2 mm^2^ in the better eye (BE) and 3.1 ± 1.3 mm^2^ in the worse eye. The most significant associations with VRQoL scores for distance and near activities were observed in the inner lower and inner left subfields of the BE, respectively. For patients with foveal-sparing GA, the LLVA of the BE stood out as the most influential variable across all VRQoL scales.

**Conclusions:**

The study’s findings point toward the pivotal role of GA location, especially the inner lower and inner left subfields of the BE, in relation to VRQoL in GA patients. The LLVA’s importance becomes even more pronounced for foveal-sparing patients. These observations highlight the need for health care professionals to better understand the association between lesion location and patient-reported outcomes. This is critical for informing treatment decisions and refining the planning of interventional trials.

**Financial Disclosure(s):**

Proprietary or commercial disclosure may be found in the Footnotes and Disclosures at the end of this article.

Age-related macular degeneration (AMD) is a leading cause of visual impairment.^1,2^ In 2019, approximately 18.34 million United States (US) individuals aged ≥ 40 years were living with early-stage AMD, and 1.49 million were living with late-stage AMD.^3^ Visual impairment caused by AMD compromises patient mobility and functioning. A retrospective study of 1901 patients with bilateral geographic atrophy (GA) secondary to AMD found that at initial diagnosis, 71.1% did not meet the United Kingdom standard of visual acuity for driving, and 7.1% were legally blind. Within 2 years, an additional 66.7% became ineligible to drive, and 16% became legally blind.^4^ Further studies have reported that approximately one-fourth of cases of blindness in the US and United Kingdom are attributable to GA.^5,6^

Geographic atrophy refers to atrophic lesions that form on the retina due to pigment epithelium and photoreceptor degeneration.^7–9^ Lesions typically originate in the perifoveal macula and progress over time. Atrophy spreads more rapidly in the direction of the periphery of the retina than the fovea and may not involve the fovea until late in the disease.^10^ No preference has been found in the direction of spread from the center,^11^ and progression kinetics depend on the individual.^12,13^ This variability makes disease monitoring complex, as historically accepted markers of visual function in GA, such as best-corrected visual acuity (BCVA) and lesion size,^14–16^ may not directly correlate with patient impact.

For this reason, there has been a growing interest in using patient-reported outcome measures in GA. Vision-related quality of life (VRQoL) strives to capture patients’ experience of their disease, including its effects on daily functioning. Limited studies have examined the relationship between structural and functional markers and VRQoL in GA. Holm et al^17^ found that BCVA and lesion size were associated with VRQoL. A study by our group reported a link between BCVA, GA size, and low luminance visual acuity (LLVA) of the better eye (BE) and VRQoL.^15^ In contrast, Ahluwalia et al^18^ found no association between the total area of atrophy in the BE or worse eye (WE) and VRQoL. When examining the location of atrophy, involvement of the central 1-mm-diameter zone of the BE was associated with lower VRQoL.

More research is needed to evaluate the relevance of structural and functional determinants of GA to VRQoL, especially in light of recent approval by the US Food and Drug Administration of drugs to slow down the progression of GA secondary to AMD.^19–21^

To our knowledge, this paper is the first to analyze the relative contribution of structural and functional markers of GA to VRQoL in individuals with AMD.

## Methods

### Patients

Patients were recruited at the Department of Ophthalmology at the University of Bonn, Germany, in the context of the Directional Spread in Geographic Atrophy study (NCT02051998; Principal Investigator: Monika Fleckenstein). This study is a noninterventional, prospective, natural history study on GA secondary to AMD. This study adhered to the tenets of the Declaration of Helsinki and was approved by the institutional review board of the University of Bonn. Written informed consent was obtained, and participants received no stipend. To be included in this analysis, patients had to be ≥ 55 years of age at the baseline visit and have GA in both eyes. Patients with exudative neovascular AMD or any other ocular disease that could affect the assessment of the retina in the study eye were excluded.

### Clinical Assessment

This study collected data on age, sex, medical history, BCVA, LLVA, reading acuity, foveal involvement, and National Eye Institute Visual Function Questionnaire 25 (NEI VFQ-25) scores. Best-corrected visual acuity, LLVA (measured using a 2.0 log neutral density filter), and reading acuity were assessed using the ETDRS and Radner Reading Charts, respectively, and converted to the base-10 logarithm of minimal angle of resolution scale.^22,23^ The NEI VFQ-25 includes a base questionnaire with 25 items that comprise a composite score and 12 subscales addressing various aspects of vision-related functioning. Scores range from 0 to 100, with higher scores indicating better function.^24,25^ In patients with neovascular AMD, a change of 4 to 6 points is considered clinically meaningful.^26^ In our study, the questionnaire was read to patients by a study nurse to enhance the quality, accessibility, and reliability of the data.

### Imaging

After pupil dilatation using 0.5% tropicamide and 2.5% phenylephrine, patients underwent 3 types of imaging using a Spectralis HRA-OCT 2 (Heidelberg Engineering): 30° × 30° fundus autofluorescence imaging (*λ* excitation, 488 nm; *λ* emission, 500–700 nm), 30° × 30° infrared reflectance imaging (*λ*, 815 nm), and 30° × 25° spectral-domain OCT imaging (121 B-scans, ART 25).

### Image Grading

Geographic atrophy size was determined using the RegionFinder software for fundus autofluorescence and infrared reflectance, as previously described, and the annotations were performed in a semiautomatic manner.^27,28^ Two readers independently graded the images, and a third reader provided arbitration if the GA size differed by > 0.3 mm^2^ between the first 2 readers. Geographic atrophy was classified as “foveal sparing” if the atrophic lesions did not include the foveal center, and there was relative preservation of the outer retinal layers on OCT imaging subfoveally.

To assess the topographic location of GA, an ETDRS grid was placed onto the fundus autofluorescence images to assess the extent of GA within the subfields. The ETDRS grid consists of a central 1-mm-diameter zone, an inner 3-mm-diameter ring, and an outer 6-mm-diameter ring. The inner and outer rings are each divided into 4 quadrants (superior, inferior, nasal, and temporal) to make 9 subfields, including the central zone.^29,30^ The size of atrophy in the different subfields was then measured and included in the analysis.

To simplify comparisons between eyes, subfields were labeled as they appear on retinal imaging (i.e., in retinal space based on the physician’s view). Subfields in the right eye temporal and left eye nasal quadrants are referred to as the left subfields (and correspond to the patient’s left visual field). Subfields in the right eye nasal and left eye temporal quadrants are referred to as the right subfields (and correspond to the patient’s right visual field). The labeling of the vertical quadrants of the ETDRS grid remained unchanged. The superior subfields correspond to the lower visual field, and the inferior subfields correspond to the upper visual field.

### Statistical Analyses

Statistical analysis of the data was performed using R software (open-source and free software, www.r-project.org) and add-on packages lme4, glmnet, stepwise, and glmmLasso. Geographic atrophy size was square root transformed. Better and WEs were defined based on BCVA, and the Shapiro-Wilk test was used to assess variable normality. For normally distributed variables, the mean and standard deviation are presented. The median and interquartile range are presented for nonnormally distributed variables. Univariable linear regression was applied to analyze the associations between the individual determinants and the dependent variable VRQoL.

Multicollinearity (≥ 2 explanatory variables with high bivariate correlation) was evident, leading to instability in model coefficients and variable selection when using conventional multivariable least-squares regression. To address this issue, we applied least absolute shrinkage and selection operator regression with the VRQoL as the dependent variable for the cross sectional multivariable analysis at baseline. Least absolute shrinkage and selection operator regression is designed to handle multicollinearity and carries out variable selection by performing regularization and shrinking coefficient estimates toward zero. This enhances the prediction accuracy and interpretability by providing a parsimonious model (i.e., a model with few predictors).^31^

Nested cross-validation with patient-wise splits was applied to estimate the prediction accuracy of the model (outer leave-one-out cross-validation) and to optimize the tuning parameter *λ* of the least absolute shrinkage and selection operator regression (nested inner leave-one-out cross-validation). Patients were considered as a random effect.

## Results

### Cohort Description

A total of 164 eyes from 82 participants with GA were included in this analysis, with 43 females and 39 males. The mean age ± standard deviation was 77.2 ± 7.5 years of age at baseline. The average area of GA was 2.9 ± 1.2 mm^2^ for the BE (range: min, max: 0.3, 5.5; corresponds to approximately 1.11 ± 0.46 disc areas) and 3.1 ± 1.3 mm^2^ (range: 0.4, 5.4; corresponds to approximately 1.19 ± 0.50 disc areas) for the WE. The median (interquartile range) NEI-VFQ-25 composite score was 70 (25) (Table 1, compared with Fig S1, graphical abstract, available at www.ophthalmologyretina.org).

### Association of the GA Location As Determined by the ETDRS Subfields with VRQoL

**Figure 2.**
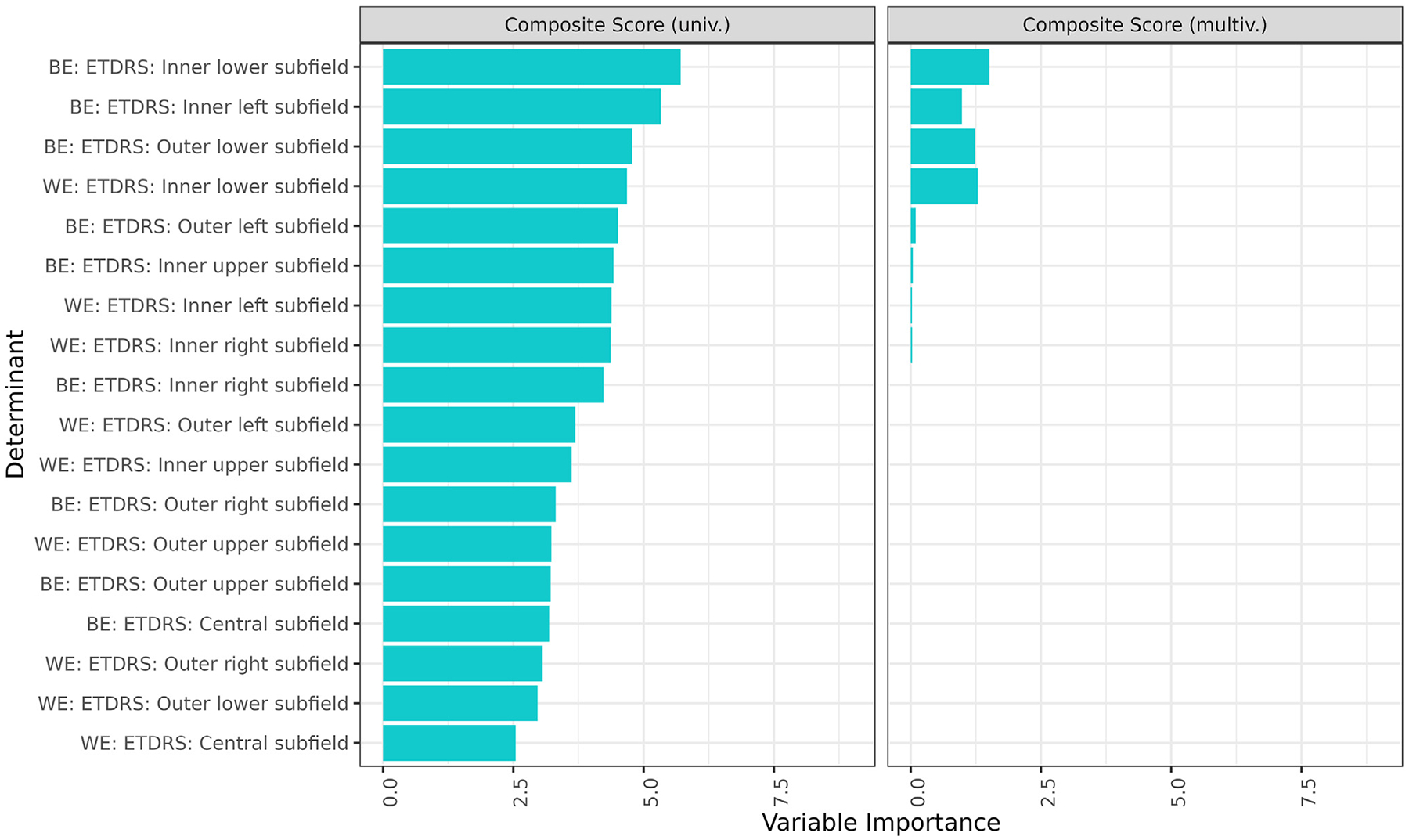
Uni- and multivariate regression of ETDRS subfields on vision-related quality of life (VRQoL) composite score. Results from univariate and multivariate regression analyses depicting the relevance of the ETDRS grid subfields to VRQoL are presented. The left columns display findings from the univariate analysis, whereas the right columns show outcomes from the multivariate analysis. It was observed that subfields from the better eye (BE) generally exhibited a stronger association with VRQoL when compared with the worse eye (WE). Particularly, the inner lower and inner left subfields of the BE emerged as most relevant. In the multivariate analysis, the inner left subfield was found to significantly contribute to the near activities subscale. The variable importance was measured by the *t* statistic of the individual univariable linear mixed-effect models and of the multivariable linear mixed-effect model with variables selected via least absolute shrinkage and selection operator regression for the univariate and multivariate analysis, respectively. Only the 10 most important variables in the univariable analysis are shown for simplicity which includes all variables selected in the multivariable analysis. multiv. = multivariate; univ. = univariate.

Univariate and multivariate regressions were performed to understand ETDRS subfields most relevant to VRQoL. Figure 2 and Figure S3 (available at www.ophthalmologyretina.org) demonstrate the results of the univariate analysis in the left columns. Subfields of the BE tended to be more highly associated with VRQoL than those of the WE. Across composite and subscale scores, the most relevant subfields were the inner lower and inner left subfields of the BE. These subfields retained significance in the multivariate, although the relative contribution of the inner left subfield to the model was greatest for the near activities subscale (Fig 2, Fig S3, right columns). For the composite score, the next most important subfields included the outer lower subfields of the BE and the inner lower subfield of the WE, which were also significant in the multivariate analysis. For the near activities subscale, the next most relevant subfield was the inner left subfield of the WE. For the distance activities subscale, these included the inner lower subfield of the WE and inner right subfield of the WE.

### Association of All Structural Determinates with VRQoL

**Figure 4.**
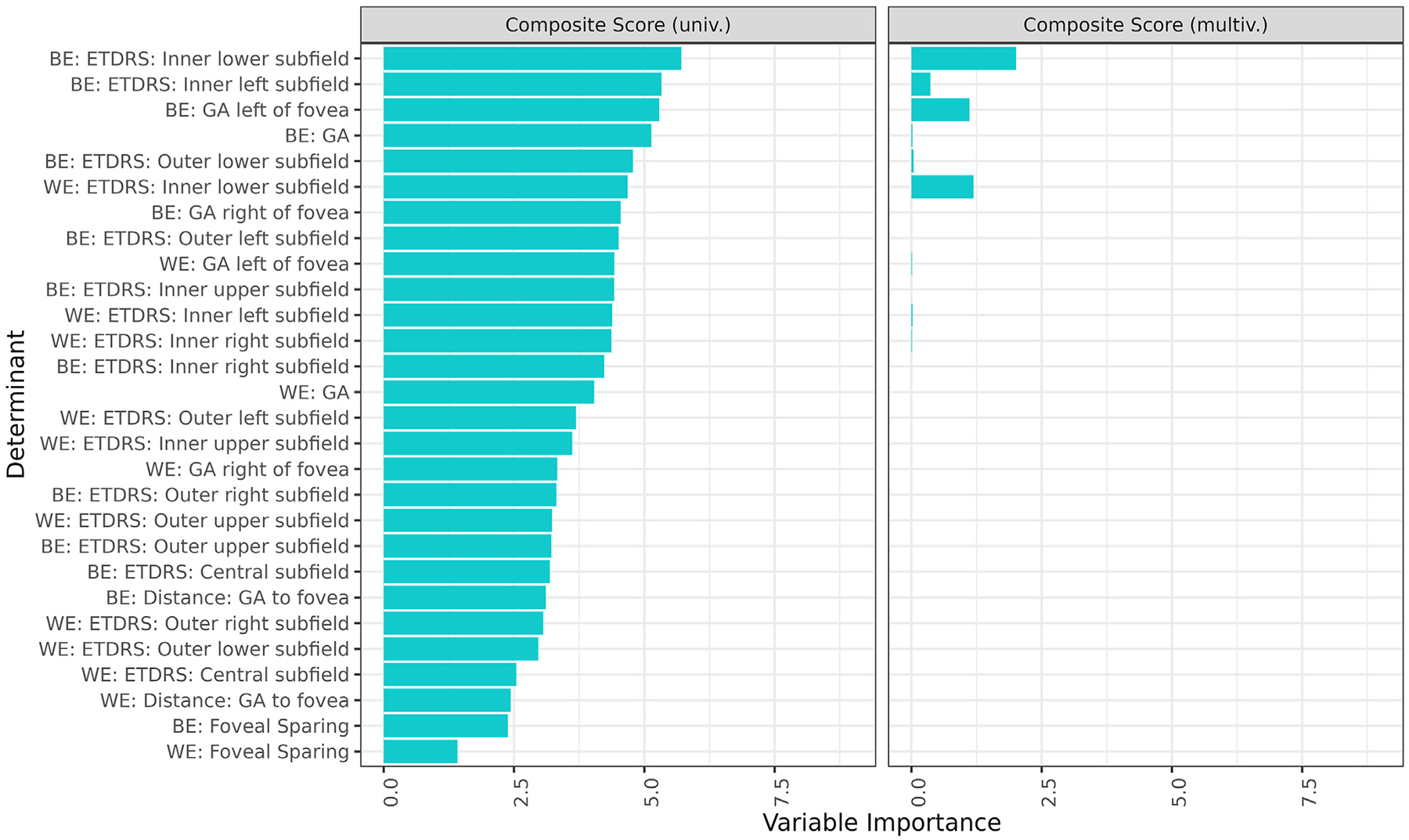
Uni- and multivariate regression of all structural determinants and ETDRS subfields with vision-related quality of life (VRQoL). Following the addition of all structural determinants, our analysis highlighted the pronounced significance of the inner lower and inner left subfields of the better eye (BE) in relation to VRQoL. These particular subfields stood out both in the univariate, as illustrated in the left column, and persisted in their relevance in the multivariate (right column). Further contributing to the composite and near activities subscales were the geographic atrophy (GA) left of the fovea and the entire area of GA in the BE. In contrast, the foveal-sparing GA of both the BE and the worse eye (WE) presented as the least pertinent variables for composite and distance activities scores. However, the univariate showed an increased relevance of foveal sparing for near activities, and it notably emerged as the dominant factor in the multivariate analysis. As for the distance activities metric, the inner lower and inner right subfields of the WE were especially noteworthy. The variable importance was measured by the *t* statistic of the individual univariable linear mixed-effect models and of the multivariable linear mixed-effect model with variables selected via least absolute shrinkage and selection operator regression for the univariate and multivariate analysis, respectively. Again, only the 10 most important variables in the univariable analysis are shown for simplicity, which includes all variables selected in the multivariable analysis. multiv. = multivariate; univ. = univariate.

All structural determinants were then added. The results underscored the importance of the inner lower and inner left subfields of the BE to VRQoL, as these again emerged as most relevant in the univariate (Fig 4, Fig S5, left column, available at www.ophthalmologyretina.org) and remained relevant in the multivariate (right column). Geographic atrophy left of fovea and total area of GA of the BE were also important to the composite and near activities subscales. Foveal-sparing GA of the BE and WE were among the least relevant variables to composite and distance activities scores; however, foveal sparing demonstrated greater salience for near activities in the univariate and was the strongest contributor to the multivariate.

### Association of All Structural and Functional Determinates with VRQoL

**Figure 6.**
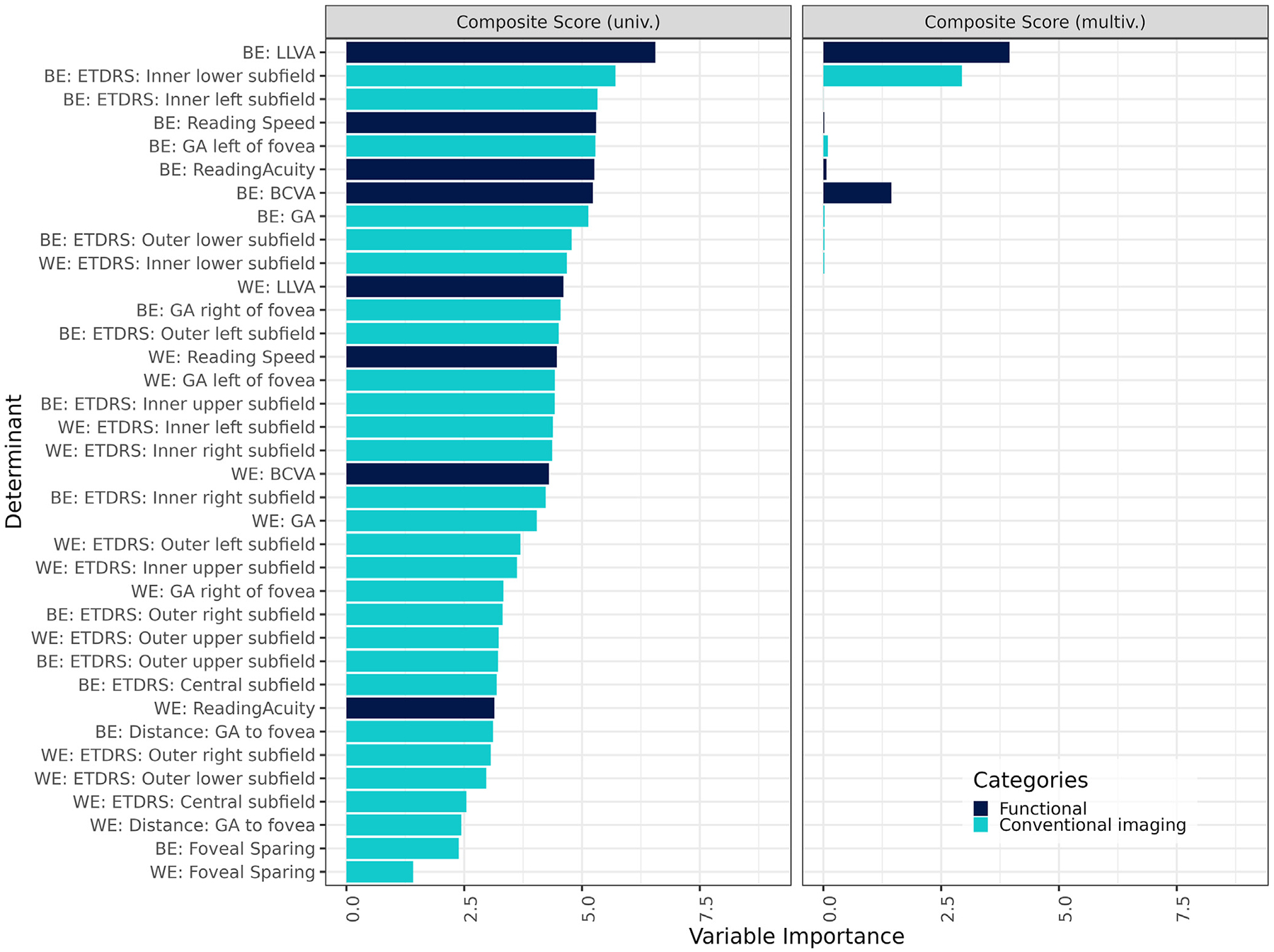
Uni- and multivariate regression of functional determinants, structural determinants, and ETDRS subfields with vision-related quality of life (VRQoL). In an effort to assess the relative impact of visual function variables on VRQoL when juxtaposed with structural elements, several key insights emerged. Within the scope of the univariate regression, the low-luminance visual acuity (LLVA) of the better eye (BE) distinguished itself as the primary factor influencing the composite scale. This was closely followed by the inner lower subfield, inner left subfield, and reading speed, all attributes of the BE. In the realm of multivariate regression, the LLVA of the BE retained its importance, consistently affecting all 3 score categories and underscoring its distinctive contribution (right column). Delving into the near activities scale, the best-corrected visual acuity (BCVA), reading acuity, and the inner left subfield of the BE surfaced as dominant variables in the univariate analysis. Meanwhile, for the distance activities scale, the inner lower subfield, LLVA, inner left subfield, and reading speed, all characteristics of the BE, established strong associations. The variable importance was measured by the *t* statistic of the individual univariable linear mixed-effect models, and of the multivariable linear mixed-effect model with variables selected via least absolute shrinkage and selection operator regression for the univariate and multivariate analysis, respectively. Again, only the 10 most important variables in the univariable analysis are shown for simplicity, which includes all variables selected in the multivariable analysis. GA = geographic atrophy; multiv. = multivariate; univ. = univariate; WE = worse eye.

Visual function variables were included to determine their comparative association with VRQoL with respect to structural factors. For a more detailed analysis of the association of various functional parameters with VRQoL, please refer to Künzel et al.^15^ In the univariate regression, LLVA of the BE was the most salient factor for the composite scale, followed by the inner lower subfield, the inner left subfield, and reading speed, all of the BE (Fig 6, Fig S7, left column, available at www.ophthalmologyretina.org). Low-luminance visual acuity of the BE remained significant for the multivariate regression across all 3 scores, demonstrating its unique contribution to the model (right column). For the near activities scale, BCVA, reading acuity, and the inner left subfield of the BE were most relevant in the univariate. For the distance activities scale, the inner lower subfield, LLVA, inner left subfield, and reading speed of the BE were highly associated.

### Association of VRQoL with the Structural and Functional Deteminates in Foveal-Sparing Patients

**Figure 8.**
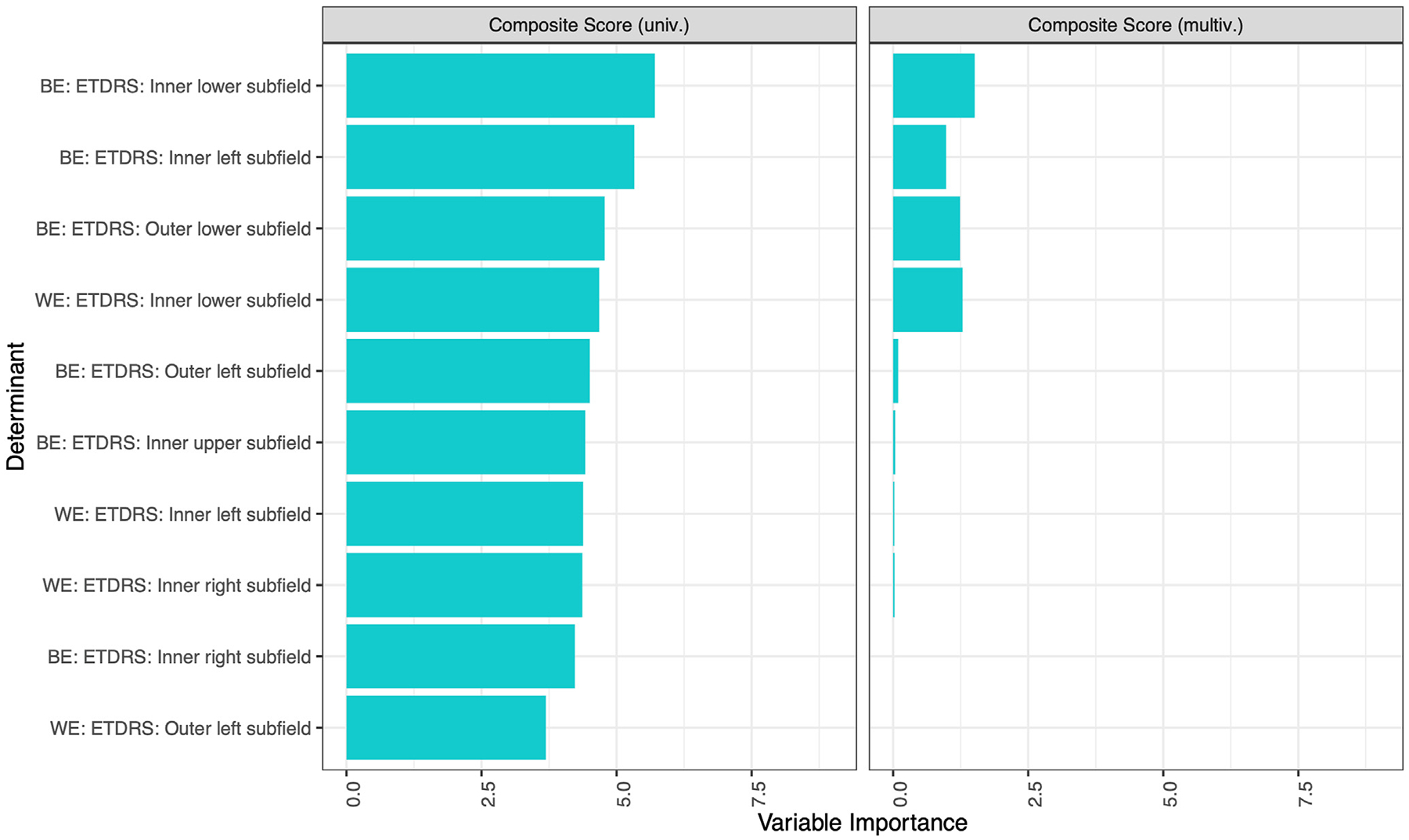
Uni- and multivariate regression of ETDRS subfields and vision-related quality of life (VRQoL) in foveal-sparing participants only. In a nuanced examination, eyes from 43 participants, with foveal-sparing geographic atrophy in ≥ 1 eye, were individually assessed. The univariate regression, restricted solely to ETDRS subfields, pinpointed the influential role of the inner lower subfield of the better eye (BE) in the composite VRQoL score. The variable importance was measured by the *t* statistic of the individual univariable linear mixed-effect models, and of the multivariable linear mixed-effect model with variables selected via least absolute shrinkage and selection operator regression for the univariate and multivariate analysis, respectively, each for the near activities (A) and distance activities (B). multiv. = multivariate; univ. = univariate; WE = worse eye.

In a final approach, 88 eyes from 44 participants with foveal-sparing GA were analyzed separately. The patients were divided into 2 groups: (1) 22 patients (44 eyes) with bilateral GA and (2) 22 patients (44 eyes) with unilateral GA. In this analysis, we included all variables of the BE and WE. The univariate regression with only ETDRS subfields demonstrated the influence of the inner lower subfield of the BE to composite and distance activities scores, as well as the inner right subfield of the BE to near activities scores (Fig 8, Fig S9, available at www.ophthalmologyretina.org) for patients with bilateral foveal sparing.

For the patients with unilateral foveal sparing, the univariate regression with only ETDRS subfields demonstrated the influence of the outer left subfield of the BE to composite, as well as the inner left and right subfields of the BE to near and distance activities scores, respectively (Fig 8, Fig S9).

**Figure 10.**
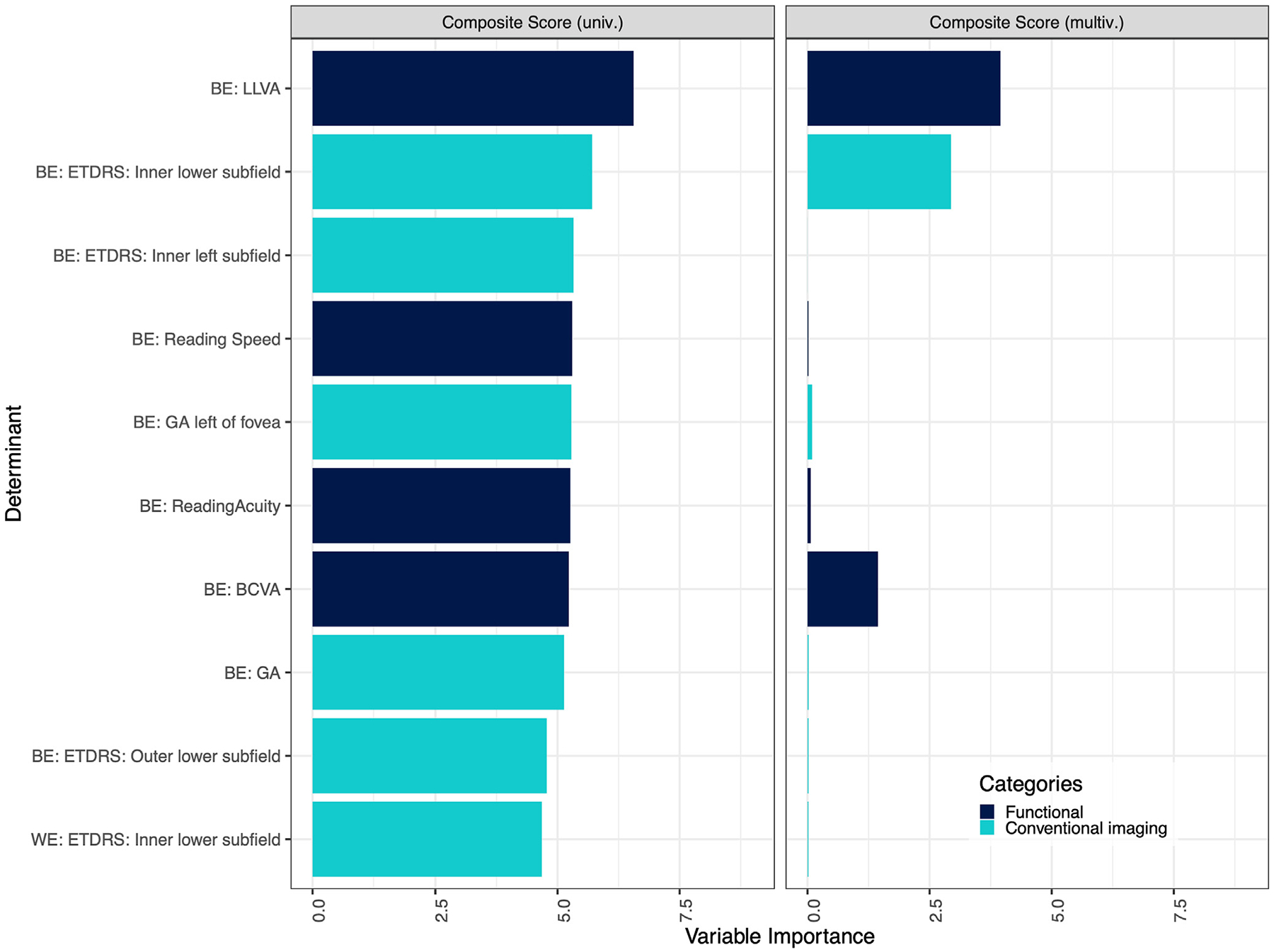
Uni- and multivariate regression of all variables and vision-related quality of life in foveal-sparing participants only. Expanding on the analysis of 43 participants with foveal-sparing geographic atrophy (GA) in ≥ 1 eye, this figure delves into the univariate regression incorporating all variables. Predominantly, the low-luminance visual acuity (LLVA) of the better eye (BE) emerges as a leading determinant for composite scores, underscoring its pivotal role. Its influence also permeates both the near activities and distance activities subscales within the foveal-sparing cohort. Although the inner lower subfield retains its centrality for the composite score, an interesting shift is observed wherein reading acuity assumes a more pronounced relevance compared with reading speed for the composite score. The variable importance was measured by the *t* statistic of the individual univariable linear mixed-effect models, and of the multivariable linear mixed-effect model with variables selected via least absolute shrinkage and selection operator regression for the univariate and multivariate analysis, respectively. BCVA = best-corrected visual acuity; multiv. = multivariate; univ. = univariate; WE = worse eye.

In the uni- and multivariate regression with all variables, LLVA of the BE once again had the greatest relevance for composite scores in the (Fig 10, Fig S11, available at www.ophthalmologyretina.org) for the patients with unilateral or bilateral foveal-sparing GA. Additionally, it emerged as significant for both the near activities and distance activities subscales in the unilateral foveal-sparing cohort, whereas the reading speed seemed more important in the bilateral foveal-sparing patients.

## Discussion

This study examined the relevance of GA determinants to VRQoL. Few studies have looked at ETDRS subfields in relation to quality of life outcomes,^18^ and to our knowledge, no prior study has shown the comparative salience of a wide range of structural and functional GA markers to different types of activities. Previously such findings had limited clinical applicability given the absence of US Food and Drug Administration-approved treatment options.

In 2023, the US Food and Drug Administration approved pegcetacoplan and avacincaptad pegol for the treatment of GA secondary to AMD. Both drugs inhibit key steps in the complement cascade and have been shown in phase III trials to reduce GA progression when administered intravitreally monthly or every other month.^19,20^ The treatment effects were rather small, and for pegcetacoplan and avacincaptad pegol, there were no significant differences in the prespecified visual function endpoints compared with sham throughout the trial periods.^19,20^ As GA is often bilateral, patients may require injections in both eyes. Moreover, treatment poses a dose-dependent risk of neovascular AMD and ischemic optic neuropathy.^32^ Less-invasive treatments, such as dietary modification,^33^ may be more suitable for certain patient populations, making it critical to understand the likely course and implications of GA location in individuals. Previous research has shown that GA favors certain patterns of progression. Geographic atrophy tends to impact center and inner ETDRS zones, with most rapid enlargement in the inner zones. Increased area of GA positively correlates with outer zone involvement, with the superior outer subfield affected more frequently than other outer subfields.^11^ Localization of GA can be a powerful tool for clinicians in making treatment determinations, as specific subfields appear to be more critical for VRQoL. A better understanding of the associations between lesion location and patient-reported outcomes can not only allow clinicians to estimate the impact of future disease progression on patients’ quality of life but also plays a crucial role in refining the planning of interventional trials.

Subfields of the BE tended to outperform their counterpart in the WE, consistent with prior studies.^15,18^ The inner lower and inner left subfields of the BE are especially important for VRQoL. When looking at structural determinants only, the inner lower subfield of the retina, corresponding to the superior visual field, was the greatest factor in the univariate and multivariate analysis for composite and distant activities scores. This subfield is implicated in activities such as driving (e.g., visualizing traffic signals), as found in the composite scale, and reading street signs or store names, as included in the distance activities subscale.

For the near activities subscale, the inner left subfield, referring to the left visual field, contributed most strongly to the univariate with structural determinants. This confirms the findings of Ahluwalia et al^18^ that GA in the left visual field was significantly associated with lower VRQoL. As Western individuals typically read from left to right, the inner left subfield may play a role in reading initiation. The left subfield has also been postulated to be important for moving to the next line while reading.^34^ Either of these functions could affect reading speed. Interestingly, although both the inner left subfield and reading speed were among the most relevant factors for the univariate regression with all variables, reading speed was no longer significant in the multivariate. Reading acuity and BCVA, by contrast, were significant to both. It is possible that GA in the inner left subfield subsumed the contribution of reading speed within the multivariate, suggesting the interrelatedness of these variables. This is supported by the study by Keuster-Gruber et al^35^ of patients with hemaniopic field defects, in which horizontal training improved reading speed to a much greater extent in individuals with left field defects than those with right. More research is needed to examine this connection.

Of the functional markers, LLVA emerged as highly salient for VRQoL. Although this was true for both samples analyzed, it was particularly evident for foveal-sparing GA. In the univariate analysis with all participants, LLVA was the most relevant variable for the composite score, while ranking slightly lower for distance activities and lower still for near activities. However, in the foveal-sparing analysis, LLVA was the most important variable for all scales in the univariate. Vision problems under low luminance are well-documented in elderly patients,^36–38^ and VRQoL related to low luminance activities is more likely to decline over time than for daytime activities.^38^ In patients with GA secondary to AMD, difficulty performing tasks under low luminance is seen at all stages of disease,^39^ and baseline deficit in LLVA is a strong predictor of future reductions in visual acuity.^40^ Balaskas et al^41^ reported that nonfoveal GA lesions were most important for LLVA prediction, highlighting their importance for future trials.^41^ Sunness et al^42^ identified adequate lighting as key for maximizing foveal vision in those with foveal-sparing scotomas. Previous studies have criticized the NEI VFQ-25 for focusing on photopic conditions to the neglect of low luminance.^38^ Even the significant role of LLVA in VRQoL illustrated in our results may be conservative due to inadequate representation of low-luminescence items. Future studies should incorporate scales targeted at low-luminescence activities^43,44^ to further investigate LLVA.

Strengths of this study were the variety of determinants used in the analysis, allowing for a holistic view of VRQoL, and the separate analysis of a foveal-sparing cohort. Limitations include the following: the results are applicable specifically to the scenario of bilateral GA as captured in this study and might differ from other GA cohorts. The sample size may limit generalizability and lack the statistical power to reveal weaker associations. Sivaprasad et al^25^ showed that in GA patients the NEI VFQ-25 composite, near activities, and distance activities scores had high internal consistency, indicating that items of the NEI VFQ-25 are highly related to each other and to the scale as a whole. However, it showed a high test-retest reliability when GA lesion size growth is low (≤ 0.45 mm^2^ over a 6-month period), highlighting its inability to capture smaller progressions.^25^ Inclusion of an additional questionnaire targeted at low-luminescence activities may have clarified the relationship between variables and VRQoL. As we described associations, further studies will be needed to analyze causal effects.

In conclusion, this study demonstrates the relative value of structural and functional GA markers to VRQoL. The inner left and inner lower subfields were most relevant for near activities and distance activities, respectively. Of the functional markers, LLVA was a notable contributor within the analysis. These findings empower clinicians to more accurately assess the disease’s real impact on patients’ quality of life and its potential influence on the progressive development of GA in different areas of the ocular fundus. A heightened understanding of the associations between lesion location and VRQoL can play a crucial role in counseling on treatment decisions and optimizing the design of interventional trials.

## Supplementary Material

Supplements

Supplemental material available at www.ophthalmologyretina.org.

## Figures and Tables

**Table 1.** Participant Demographics, GA Characteristics, and VRQoL Performance

Determinant	Mean ± SD Median (IQR)
Participants	82 (39 male, 43 female)
Age, yrs	77.2 ± 7.5
BCVA BE, logMAR	0.30 (0.48)
BCVA WE, logMAR	0.84 (0.7)
GA area BE, mm	2.9 ± 1.2
GA area WE, mm	3.1 ± 1.3
Foveal sparing at least one eye, %	52.4% (43 eyes)
Foveal sparing both eyes, %	28% (23 eyes)
Reading speed BE, WPM	92 (99)
Reading speed WE, WPM	0.0 (95)
Reading acuity BE, logRAD	0.63 (0.88)
Reading acuity WE, logRAD	1.3 (0.69)
VRQoL composite score	70 (25)
VRQoL near activities score	50 (33)
VRQoL distance activities score	58 (33)

The cohort characteristics are shown. For normally distributed variables, the mean ± SD are shown. For nonnormally distributed variables, the median and IQR are given.

BCVA = best-corrected visual acuity; BE = better eye; GA = geographic area; IQR = interquartile range; logMAR = logarithm of the minimum angle of resolution; logRAD = logarithm of the reading acuity determination; SD = standard deviation; VRQoL = vision-related quality of life; WE = worse eye, WPM = words per minute.

## Abbreviations and Acronyms:

AMD: age-related macular degeneration
BCVA: best-corrected visual acuity
BE: better eye
GA: geographic atrophy
LLVA: low-luminance visual acuity
NEI VFQ-25: National Eye Institute Visual Function Questionnaire 25
US: United States
VRQoL: vision-related quality of life
WE: worse eye
