# Supplementary material for: Association of Lesion Location and Functional Parameters with Vision-Related Quality of Life in Geographic Atrophy Secondary to Age-related Macular Degeneration
Source: Ophthalmol Retina. Author manuscript; Available in PMC 2026 Feb 4. (PMC12872259; doi:10.1016/j.oret.2024.01.025)
Supplement: Supplements [file NIHMS2142179-supplement-Supplements.zip › 1-s2.0-S2468653024000575-mmc2.docx]

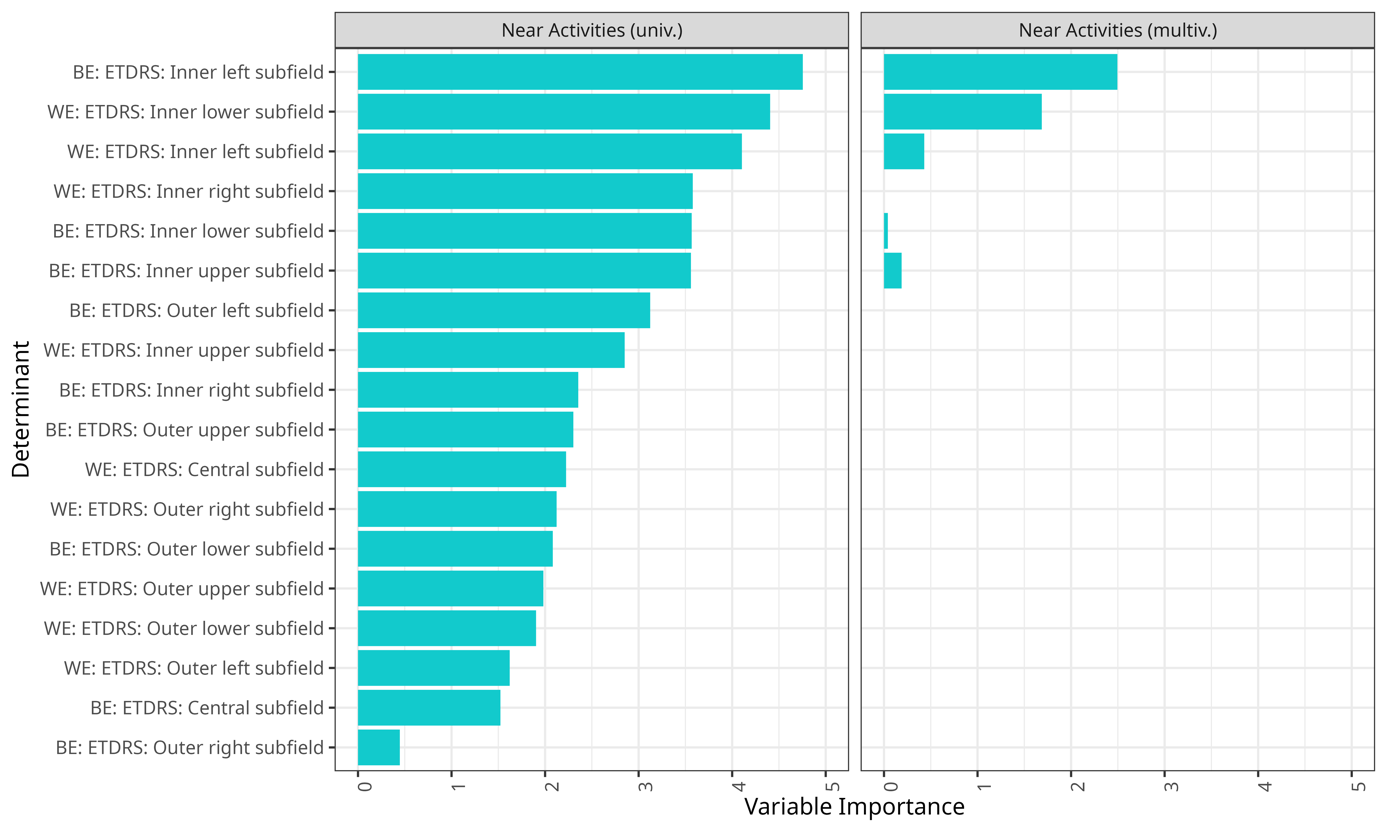
**A**


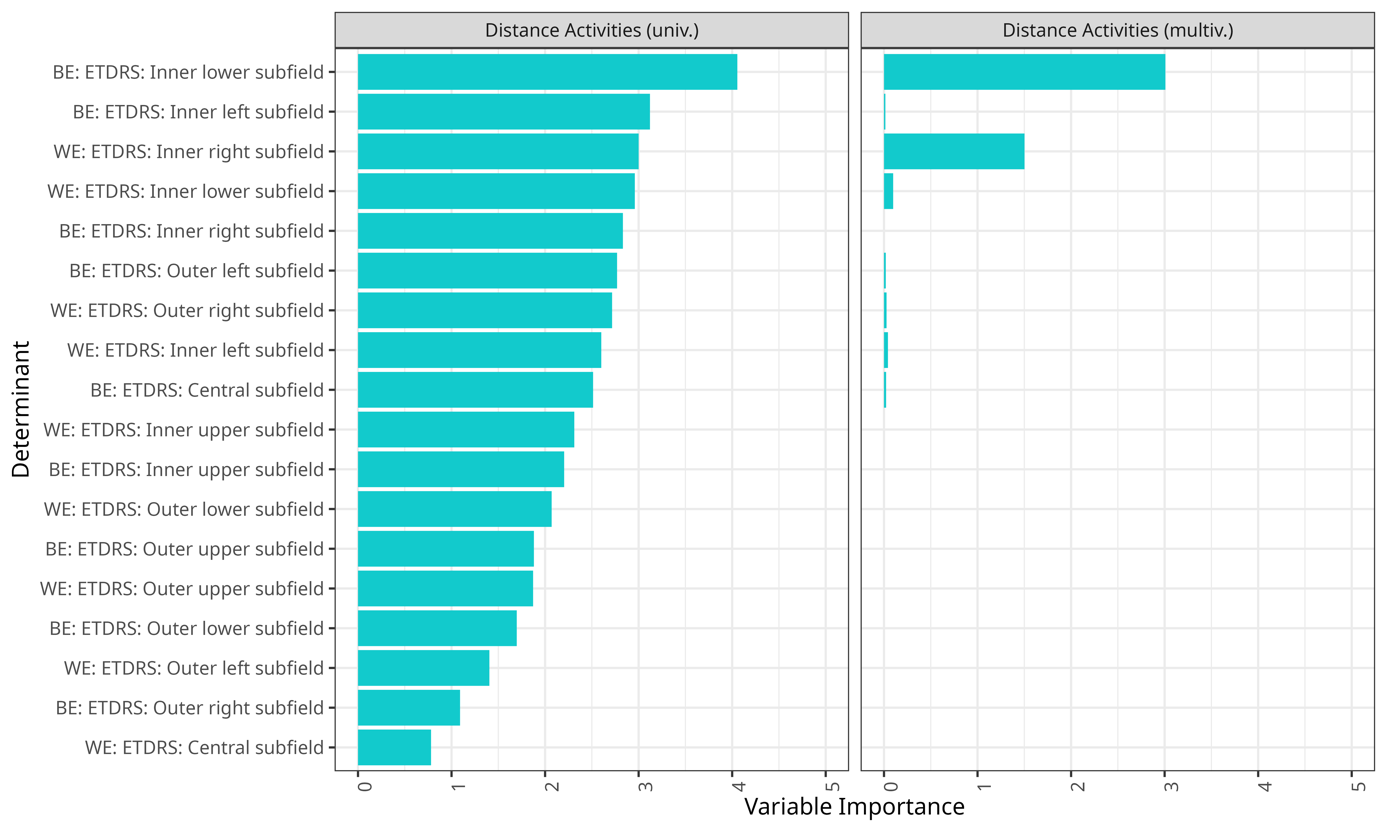
**B**

**Figure S9: Uni- and multivariate regression of ETDRS subfields and VRQoL in foveal-sparing participants only for near and distance activities.**

The variable importance was measured by the *t* statistic of the individual univariable linear mixed-effect models, and of the multivariable linear mixed-effect model with variables selected via least absolute shrinkage and selection operator regression for the univariate and multivariate analysis, respectively, each for the near activities (A) and distance activities (B). BCVA, best-corrected visual acuity; BE, better eye; ETDRS, Early Treatment Diabetic Retinopathy Study; GA, geographic atrophy; LLVA, low luminance visual acuity; WE, worse eye
