# Supplementary material for: Association of Lesion Location and Functional Parameters with Vision-Related Quality of Life in Geographic Atrophy Secondary to Age-related Macular Degeneration
Source: Ophthalmol Retina. Author manuscript; Available in PMC 2026 Feb 4. (PMC12872259; doi:10.1016/j.oret.2024.01.025)
Supplement: Supplements [file NIHMS2142179-supplement-Supplements.zip › 1-s2.0-S2468653024000575-mmc1.pdf]

Supplementary Material:

Impact of lesion location and functional parameters on vision-related quality of life in geographic atrophy secondary to AMD

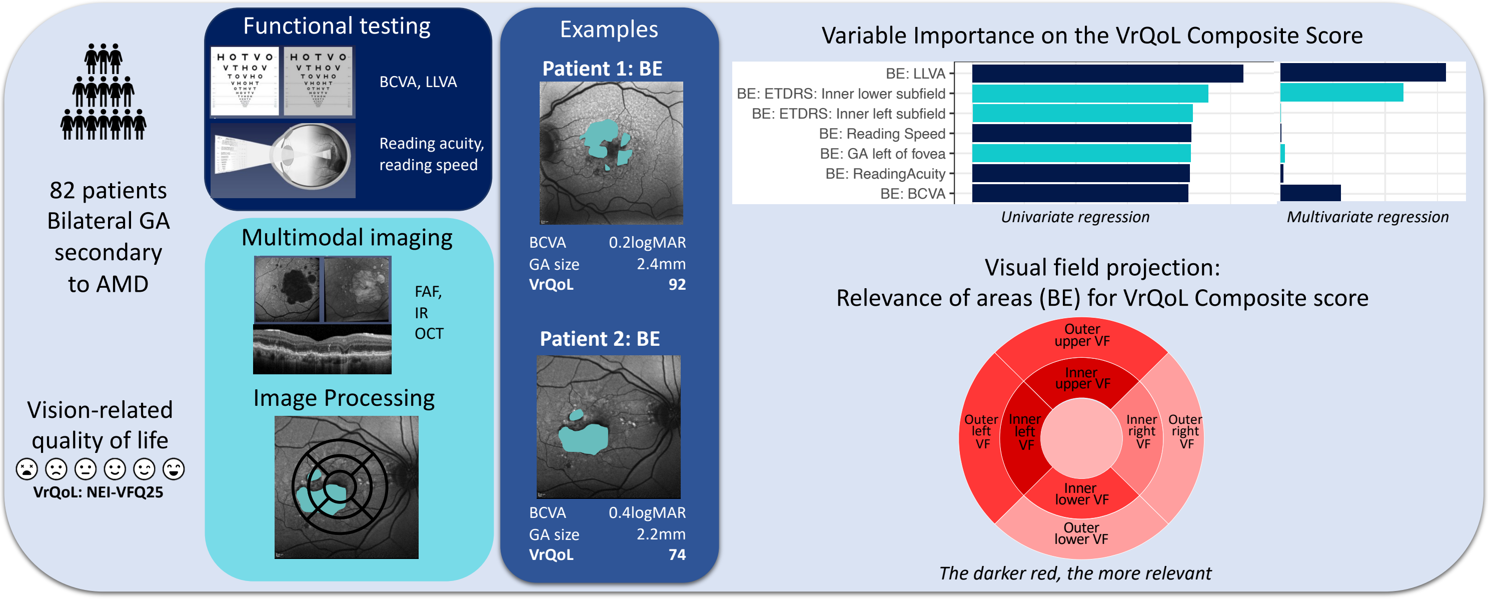

VrQoL: Vision related Quality of life, BCVA: best-corrected visual acuity, LLVA: Lo-luminance visual acuity; BE: better eye; GA: Geographic atrophy; AMD: age-related macular degeneration

Supplementary Figure S1: Graphical Abstract

This is a graphical representation that summarizes the main content and key findings of the study in a visually appealing and easily understandable manner.
